# Impact of Nano-Crystalline Diamond Enhanced Hydrophilicity on Cell Proliferation on Machined and SLA Titanium Surfaces: An In-Vivo Study in Rodents

**DOI:** 10.3390/nano8070524

**Published:** 2018-07-13

**Authors:** Robert Gerhard Stigler, Kathrin Becker, Michela Bruschi, Doris Steinmüller-Nethl, Robert Gassner

**Affiliations:** 1 Department of Oral and Maxillofacial Surgery, Medical University Innsbruck, 6020 Innsbruck, Austria; robert.stigler@i-med.ac.at (R.G.S.); bruschi.michela@gmail.com (M.B.); Robert.gassner@tirol-Kliniken.at (R.G.); 2 Department of Orthodontics, Universitätsklinikum Düsseldorf, 40225 Düsseldorf, Germany; 3 DiaCoating GmbH, 6112 Wattens, Austria; doris.steinmueller@diacoating.com

**Keywords:** cell proliferation, soft-tissue healing, chemical surface modification, surface treatment, hydrophilicitiy, nano-crystalline diamond coating

## Abstract

By coating surfaces with nano-crystalline diamond (NCD) particles, hydrophilicity can be altered via sidechain modifications without affecting surface texture. The present study aimed to assess the impact of NCD hydrophilicity on machined and rough SLA titanium discs on soft tissue integration, using a rodent model simulating submerged healing. Four different titanium discs (machined titanium = M Titanium, NCD-coated hydrophilic machined titanium = M-O-NCD, sand blasted acid etched (SLA Titanium) titanium, and hydrophilic NCD-coated SLA titanium = SLA O-NCD) were inserted in subdermal pockets of 12 Wistar rats. After one and four weeks of healing, the animals were sacrificed. Biopsies were embedded in methyl methacrylate (MMA), and processed for histology. The number of cells located within a region of interest (ROI) of 10 µm around the discs were counted and compared statistically. Signs of inflammation were evaluated descriptively employing immunohistochemistry. At one week, M-O-NCD coated titanium discs showed significantly higher amounts of cells compared to M Titanium, SLA Titanium, and SLA-O-NCD (*p* < 0.001). At four weeks, significant higher cell counts were noted at SLA-O-NCD surfaces (*p* < 0.01). Immunohistochemistry revealed decreased inflammatory responses at hydrophilic surfaces. Within the limits of an animal study, M-O-NCD surfaces seem to stimulate cell proliferation in the initial healing phase, whereas SLA-O-NCD surfaces appeared advantageous afterwards.

## 1. Introduction

Dental implants are frequently used to achieve an oral rehabilitation of partly or completely edentulous patients. Whereas several surface modifications and chemical treatments have been identified to improve osseointegration [1], the creation of an ideal transmucosal part is still challenging. On the one hand, the formation of a connective-tissue seal through collagen adhesion is desired to prevent bacterial penetration, thus favoring rough surfaces. On the other hand, as rough surface promotes biofilm adhesion [2], machined surfaces may be beneficial to prevent peri-implant inflammation.

In recent years, various mechanical and chemical surface treatments have been proposed to improve hard and soft tissue adhesion [3,4,5,6]. At the time being, titanium still is the most commonly used material for dental implants and abutments, and altering the surface roughness or hydrophilicity are common techniques to enhance both osseointegration as well as soft tissue adhesion [7,8,9].

In contrast to the common surface modifications, recent findings suggest that the alteration of titanium surface hydrophilicity through nano-crystalline diamond coating (NCD) [10] may have anti-bacterial properties [11], while promoting soft tissue adhesion [12].

As nanocrystalline particles have a size of 5 to 15 nm [13], these coatings produce a nanoscale roughness below 15 nm. Hydrophilicity is altered by saturating the dangling bonds of the NCD coating with either hydrogen (H-) or oxygen (O-), thus generating either hydrophilic (O) or hydrophobic (H) surfaces [13].

These O- and H-NCD coatings have been applied to machined titanium (M Titanium) surfaces within in vitro as well as in vivo studies, and O-NCD seemed to have improved cell attachment [12,14,15]. Bacterial adhesion was reported to be lower at O-NCD coated surfaces compared with conventional stainless steel and titanium surfaces [16].

However, it is not yet clear if the O-NCD coatings last for the entire healing phase, or if the positive effects are limited to the initial phase. In addition, it remains to be discovered if O-NCD sand blasted/acid-etched titanium (SLA Titanium) surfaces can further enhance soft tissue attachment compared with M-O-NCD.

Hence, the aim of the present investigation was to apply the novel O-NCD coatings to SLA (SLA-O-NCD) and machined titanium (M-O-NCD) surfaces and compare the respective surfaces with the untreated SLA Titanium and m Titanium controls in a rodent model.

## 2. Materials and Methods

### 2.1. Creation of Titanium Discs with Different Surface Modifications

**Figure 1:**
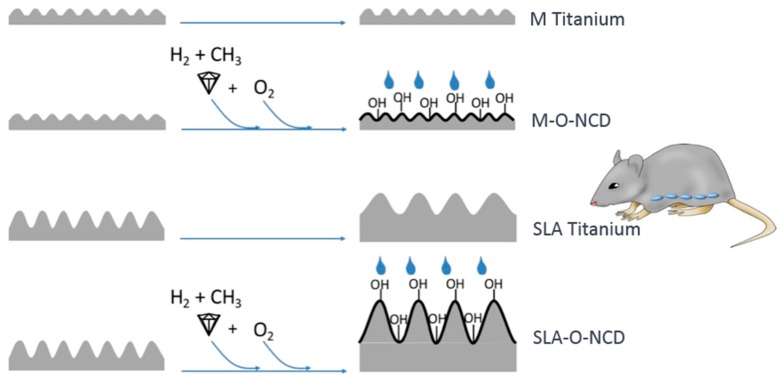
Study design showing the four different surfaces with different levels of roughness (machined versus sand-blasted/acid etched) and different hydrophilicity achieved through additional nano-crystalline diamond coating.

A total of 96 titanium discs with a diameter of 5 mm (Straumann AG, Basel, Switzerland) were used. One half (48 discs) had a machined titanium (M Titanium) surface with a roughness of 0.5 µm, and the remaining 48 discs had a sand blasted/acid-etched (SLA Titanium) surface with a roughness of 2.0 µm (Figure 1).

A total of 24 M Titanium and 24 SLA Titanium discs were subject of hydrophilic nano-crystalline diamond (O-NCD) coating using a modified hot-filament chemical vapour deposition technique [10]. In brief, a 3% CH_4_ gas diluted in hydrogen at high temperatures (720 °C) and a distinctpressure (5 mbar) were applied to the surfaces. This procedure created a NCD layer (average grain size: 5 nm), which did not alter the surface topography and provided a very high sp^3^ hybridisation of the carbon [10].

A total of 24 NCD-coated M Titanium and 24 NCD-coated SLA Titanium discs were processed for altered hydrophilicity: By heating NCD at 400 °C with an aeration of 21% oxygen for 4 h, the terminal hydrogen groups were replaced by oxygen groups, resulting in an increased hydrophilicity (hydrophilic nano-crystalline diamond O-NCD; contact angle <10°) [10]. In contrast to these modifications, pure titanium usually provides an intermediate hydrophilic or hydrophobic property with a contact angle of 70° [15,17].

In summary, two groups of M Titanium (uncoated machined titanium surface (M Titanium), and machined NCD-coated hydrophilic titanium surface (M-O-NCD)) and two groups with an SLA surface topography (uncoated SLA titanium (SLA Titanium), SLA and NCD coated hydrophilic titanium (SLA-O-NCD)) were used for the present investigation.

### 2.2. Animal Experiment

A total of 24 female Wistar rats (Charles River Laboratories, 160–210 g) was used. The trial was approved by the local authorities and permission was granted by the Austrian government (BMWF-66.011/0095-C/GT/2007). The study was designed and conducted according to the ARRIVE guidelines for experimental studies [18].

The rats were kept in groups of four in cages at the central laboratory animal facility of the medical university of Innsbruck and were provided with food and water *ad libidum*. After 4 weeks of adaption, surgeries were performed by one experienced maxillofacial surgeon. Subcutaneous implantations of the discs were performed in general anaesthesia with Ketasol (100 mg/kg body weight) (Dr. E. Graeub AG, Bern, Switzerland) and Xylasol (10 mg/kg body weight) (Dr. Graub AG, Bern, Switzerland): First the abdominal wall was shaved and the rats’ skins were disinfected with Octenisept (Schuelke, Germany). After skin incision, four subdermal pockets were created within each animal, respectively. One disc of each group was inserted per subcutaneous pockets such that the modified surface faced the dermis. The discs were allocated to the pockets at random according to a computer-generated list. A minimum distance of 1 cm was strictly kept between the pockets.

The defects were sutured tightly using resorbable material (Vicryl, Ethicon, Johnson & Johnson USA). Postoperative analgesia was performed with 4 mg/kg body weight Carprofen (Rimadyl, Pfitzer Animal Health, Berlin, Germany).

From each group, twelve animals were killed by Narcoren injection (Merial GmbH, Hallbergmoos, Germany) at one and four weeks, respectively. Thus, 12 × 4 biopsies were available at each time point.

### 2.3. Histological Evaluation

The biopsies were fixed in 4% paraformaldehyde. After a series of dehydration steps using increasing ethanol concentrations, the samples were embedded in Technovit 9100 NEU (Kulzer Department, Heraeus-Kulzer, Wehrheim, Germany) following the manufacturer’s instructions. The biopsies were sawed at the central aspect using a microtome and two samples per group were ground to a final thickness of 5 µm as described previously. One half was processed for immunohistochemistry and the other half for van Giesson’s staining. The van Giesson’s staining allows the visualizing of collagen and cell nuclei, enabling a detailed interpretation of the cellularity and collagenous network adjacent to the implant surface.

Two independent investigators (R.G.S., M.K.) assessed the cell numbers at the implant surface using a Nikon Eclipse 801 microscope (Nikon GmbH, Wien, Austria) and the manufacturer software (NIS elements BR 3.0). Therefore, all nuclei in a 10 µm distance to the surface were counted within the region of interest (ROI, Square defined as 10 µm × 130 µm manually with a 1000× magnification) and the collagen orientation and scar formation was evaluated on two different randomly chosen sections of each disc.

### 2.4. Immunohistochemistry

To evaluate proliferation, inflammatory response, and cell adhesion, immunohistochemistry was performed. Proliferating nuclear antigen (PCNA, clone PC10, mouse IgG, Chemi-Con, Billerica, MA, USA) served as a marker for proliferation, tumor necrosis factor alpha (TNF-alpha, clone M-18, goat IgG, Santa Cruz Biotechnology, Santa Cruz, CA, USA) for inflammation and Fibronectin (FBN, clone C-20, goat IgG Santa Cruz Biotechnology, Santa Cruz, CA, USA) for cell–matrix interactions. After dissolving of Technovit 9100 NEU and antigen retrieval with trypsin (Sigma Aldrich, St. Louis, MO, USA), the antibodies were applied for 10 h. For mouse antibody detection, the Dako envision system (Dako, Glostrup, Denmark) was used according to the manufacturer’s protocols. As secondary antibody to the TNF-alpha and FBN staining, an anti-goat antibody (Sc 3851; Santa Cruz Biotechnology, Santa Cruz, CA, USA) was applied.

### 2.5. Statistical Analysis

Statistical analysis was performed with SPSS (SPSS Version 24, SPSS Inc., Chicago, IL, USA). Descriptive parameters were assed for each variable and groups. A between-group comparison was achieved using the Kruskal-Wallis test. If the Kruskal-Wallis test revealed a significant difference pairwise testing was performed, the Wilcoxon signed rank test was used. *p*-values were adjusted using the Bonferroni correction for multiple tests. Results were found significant at *p* < 0.05.

## 3. Results

During the experimental phase of the study, postoperative healing was considered uneventful. Neither local or generalized infections nor allergic reactions were noted.

### 3.1. Histological Analysis

**Figure 2:**
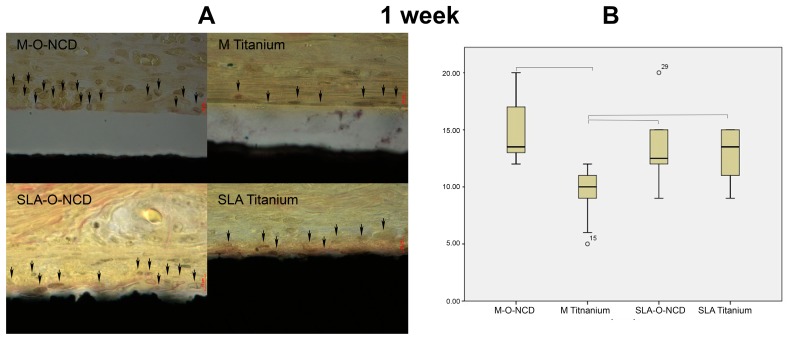
Van Giesson staining (**A**). After 1 week, more cells (black arrows) were seen in proximity to the machined hydrophilic surface compared with the other surfaces. The red scale bar indicates a 10 µm distance. Graph (**B**) shows the number of cells within a 10 µm distance to the surface after 1 week. Machined hydrophilic surfaces attracted the most cells, but significant differences were also observed between M Titanium and the other groups.

**Figure 3:**
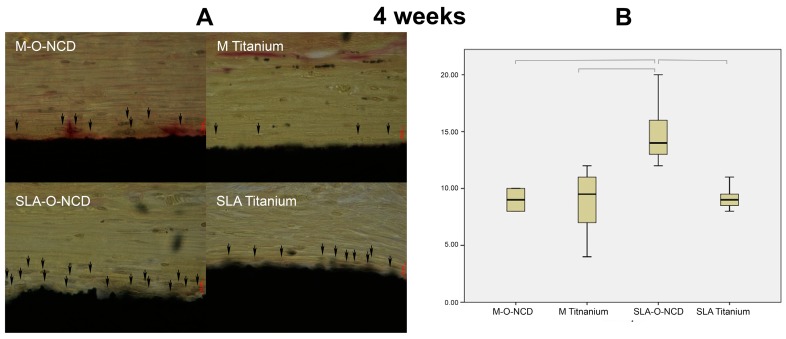
Van Giesson staining (**A**) revealed most cells in proximity to the rough hydrophilic nano-crystalline diamond coated surface. The red scale bar indicates a 10 µm distance. Boxplot (**B**) shows the descriptive values, and also highlights significant differences through bars.

Histological images were obtained at one and four weeks of healing (Figure 2 and Figure 3). The highly magnified histologic pictures showed the cell nuclei of adhering cells and the collagen fibers that are orientated parallel to the samples surface.

At one week of healing, the Kruskal-Wallis test showed significant differences in cell counts among the groups (*p* < 0.001), so multiple group comparison testing was performed. M Titanium (9.66 ± 2.22 cells/ROI) showed significant less cell counts compared with the remaining groups (M-O-NCD: 14.7 ± 2.75 cells/ROI, *p* < 0.001; SLA Titanium 13.07 ± 2.06 cells/ROI; *p* = 0.009; SLA-O-NCD: 13.5 ± 3.25 cells/ROI; *p* = 0.028). Significance failed for pairwise-comparisons among M-O-NCD, SLA-O-NCD and SLA Titanium groups.

At 4 weeks of healing, the Kruskal Wallis test revealed significant differences among the groups (*p* = 0.007), and the post hoc test yielded significantly higher cell counts for SLA-O-NCD surfaces (15.0 ± 3.16 cells/ROI), compared with SLA Titanium (9.13 ± 0.99 cells/ROI, *p* < 0.014), M-O-NCD (8.87 ± 2.75 cells/ROI, *p* < 0.018), and M Titanium (8.8 ± 2.75 cells/ROI, *p* < 0.027).

In general, at week 4, the collagen fiber bundles were orientated parallel to the surfaces at all surfaces investigated. The SLA O-NCD surfaces were marked by higher cell counts in the collagenous network compared to the M Titanium, M-O-NCD, and SLA Titanium group.

### 3.2. Immunohistochemistry

**Figure 4:**
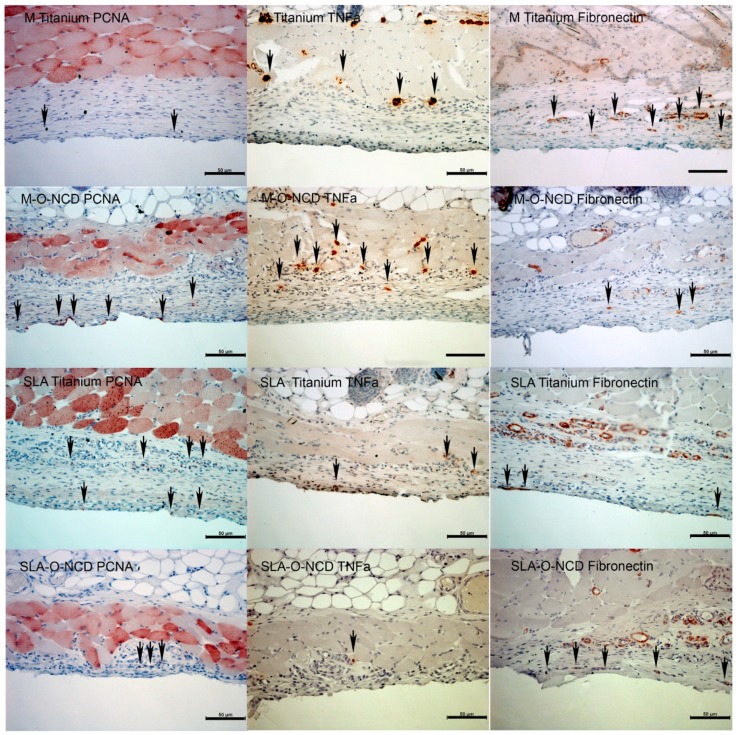
Immunohistochemistry after 1 week. PCNA showed proliferating cells in all samples, especially on the hydrophilic machined surface. The inflammatory protein TNFa was found near the SLA Titanium surface and not near hydrophilic layers. In deeper layers, TNFa expression was seen in all samples, serving as staining control. Fibronectin was mainly expressed on the rough hydrophilic surface. As fibronectin is usually found in vessels, these signals served as staining control. Scale bars indicate 50 µm.

At week one of healing, proliferating cells, detected by proliferating cell nuclear antigen (PCNA) expression, were found in proximity to all titanium surfaces. PCNA detection was highest near the M-O-NCD surfaces (Figure 4). Surfaces close to expression sites of TNFa were located at the soft tissue: the SLA Titanium interface. TNFa activity was also found in deeper layers in M-O-NCD and M Titanium samples. Fibronectin appeared to be increased on all SLA surfaces.

**Figure 5:**
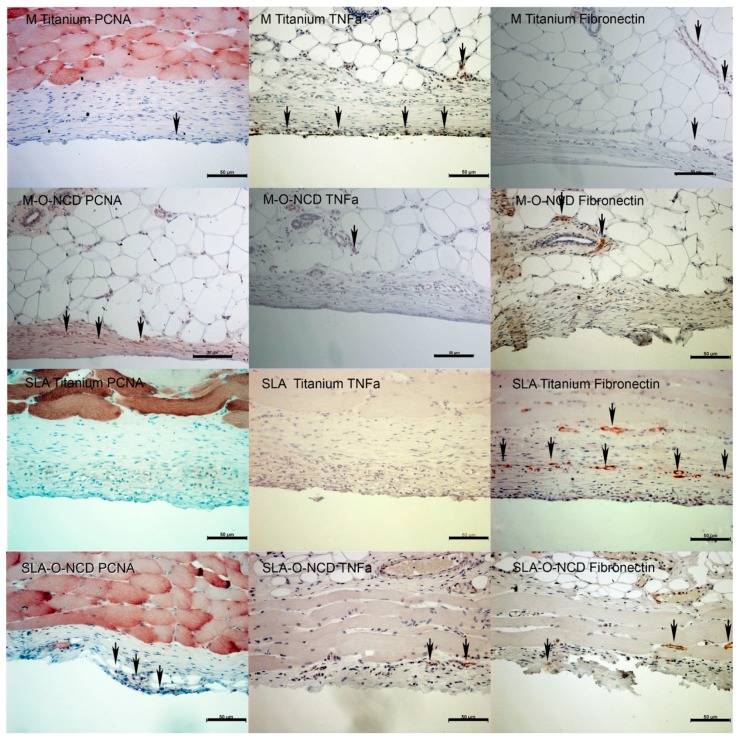
Proliferation and near-surface expression of fibronectin was only seen on the SLA-O-NCD titanium plate. Other positive fibronectin signals belong to surrounding vessels. TNFa was upregulated only for the machined titanium surfaces. Scale bars indicate 50 µm distance.

After 4 weeks of healing, lower amounts of PCNA activity were detected, whereas M-O-NCD and SLA-O-NCD surfaces presented higher PCNA expression compared to the other groups (Figure 5).

TNFa activity was found at the uncoated M Titanium surfaces (Figure 5). Fibronectin expression close to the soft tissue sample interface was only observed in the SLA-O-NCD group, whereas in deeper layers, Fibronectin detection in the vasculature served as a positive control.

## 4. Discussion

The aim of the present investigation was to assess if nano-crystalline diamond (NCD) coatings promote soft-tissue adhesion both in the early healing phase and at four weeks after surgery, and to analyse if the effect of hydrophilic NCD depends on surface roughness, thus comparing SLA to machined surfaces with and without hydrophilic NCD.

Altering surface texture may enhance the biological response on implant materials. In bone tissues, rougher titanium surfaces were commonly associated with increases in cell proliferation and differentiation, and thus with higher bone-to-implant contact values [12]. The beneficial effect of surface roughness on soft tissue attachment, however, is controversially discussed because increased roughness also favors biofilm adhesion.

Thus, hydrophilic surfaces which have sufficient micro-roughness to promote cell proliferation and adhesion, but exhibit anti-bacterial properties at the same time are demanded. According to the literature, an antibacterial effect has been reported for O-NCD coatings [16], whereas other studies reported on improved cell-adhesion associated with hydrophilic surfaces [19]. Hence, the demanded features may be associated with O-NCD coatings, and this hypothesis was supported by the findings of the present investigation:

Significant higher cell counts were detected around M-O-NCD (week 1) and SLA-O-NCD (week 4) surfaces compared with the non-coated groups, thus favoring O-NCD coated surfaces over the control groups.

A possible explanation for the higher cell counts on M-O-NCD surfaces in week 1 and SLA-O-NCD surfaces at week 4 can be derived from a recent finding reporting that within the first 3 h, most of the fibroblasts attach to polished surfaces, whereas after 3 days of healing more cells are found close to rough surface textures [20]. This phenomenon has also been described by Kim et al., who found surface roughness to significantly enhance the connective tissue attachment [21].

Beside cell counts, the functional parameters fibronectin (FBN), proliferating cellular antigen (PCNA) and tumor necrosis factor alpha (TNFa) expression were assessed immunohistochemically. In the present study, fibronectin, a protein for cell adherence, was mainly expressed close to SLA-O-NCD and SLA Titanium surfaces. This finding is in-line with Schwarz et al., who reported on elevated fibronectin expression at micro-rough surfaces over time but found no differences in fibronectin expression between the SLA and modified SLA groups. This is comparable with the results of the present study where fibronectin was found on rough uncoated SLA and SLA-O-NCD surfaces, although higher expression levels were associated with O-NCD treatment.

The proliferation rate of cells near the surface was evaluated via PCNA expression. A clearly enhanced PCNA expression was observed at all O-NCD coated surfaces. The high expression levels of PCNA on O-NCD coated polished titanium surface was already described by Kloss et al. [12]. The present work revealed that finding remains true for O-NCD coated rough surfaces.

As high cell numbers—especially with increased nucleus or plasma relations—may result from inflammatory reactions, the antibody for tumor necrosis factor alpha (TNFa) was employed in the present investigation. At one week of healing, the TNFa signals were strongest on the uncoated SLA Titanium surfaces, whereas no TNFa signals were detected on the two hydrophilic surfaces. At 4 weeks, TNFa expression was only present at M Titanium surfaces. This suggests that the agglomerated cells on the O-NCD titanium and the M-O-NCD surfaces perhaps do not result from inflammatory processes, and that hydrophilic surfaces may be of higher biocompatibility.

For the clinician, the time point of cell adhesion appears relevant. As current clinical concepts discuss an early to immediate loading of dental implants, the early formation of a stable soft-tissue seal is of great importance [22]. According to the findings of the present investigation and with the limits of a study in rodents, early cell proliferation may be promoted by hydrophilic O-NCD coatings.

These limitations suggest that the present model did not account for potential differences between dermal and gingival fibroblasts. Nonetheless, the fundamental characteristics between dermal and gingival fibroblasts were found comparable and only 5% of the genes were reported to differ [23]. The strict and standardized design of the present investigation focused on the soft tissue interaction. Thus, it demanded an inbreed stem model, where all additional bias from biofilm and functional loading was excluded. To provide reasonable sample dimensions and acceptable distances between the samples, the subdermal pocket model was selected. Nonetheless, whether the early preference for M-O-NCD and the later beneficial effect of SLA-O-NCD remain valid in clinical practice remains to be investigated in future studies.

If the results from the present investigation remain valid in humans, the O-NCD coated surfaces may also be associated with higher biocompatibility and decreased risk of biofilm adhesion.

Despite of the promising findings of the present investigation, it remains to be discovered how the O-NCD coated surfaces behave under regular stresses from chewing forces in the mouth, in the presence of oral bacteria and in the presence of specific nutritional habits and with different qualities of oral hygiene. In this study, an inbreed rat-stem animal model was used, as previously described by Kloss and co-workers [12]. The experimental setup was strictly focused on soft tissue interaction under standardized conditions, comparable to the Macaca model published by Lee [24]. By using the submerged healing, additional sources of bias from biofilms or functional loading were excluded [25]. These factors may promote development of peri-implant inflammation and may also disturb the formation of a soft tissue seal. As the soft tissue surface interface is a crucial part in the prevention of peri-implantitis, the interaction of surface topography and hydrophilicity on cell adhesion can be more clearly investigated using this submerged design study in an animal model.

## 5. Conclusions

Within the limitations of an animal study, the present study revealed that cell proliferation may be promoted by O-NCD coatings at machined and SLA surfaces. It also indicated that M-O-NCD may be beneficial in the initial healing phase, when cell attraction and proliferation are of greatest importance, whereas SLA-O-NCD may be advantageous in the subsequent healing phases. Further effects associated with hydrophilicity seem to be a greater biocompatibility and antibacterial properties. Whether these findings can be translated to humans remains to be investigated in larger animal models and randomized clinical trials.

## 6. Patents

Doris Steinmüller-Nethl and Robert Gassner hold a patent on the coating process.

## References

[1] Van Velzen F.J., Ofec R., Schulten E.A., Ten Bruggenkate C.M. (2015). 10-year survival rate and the incidence of peri-implant disease of 374 titanium dental implants with a SLA surface: A prospective cohort study in 177 fully and partially edentulous patients. Clin. Oral Implants Res..

[2] Subramani K., Jung R.E., Molenberg A., Hammerle C.H. (2009). Biofilm on dental implants: A review of the literature. Int. J. Oral Maxillofac. Implants.

[3] Esfahanizadeh N., Motalebi S., Daneshparvar N., Akhoundi N., Bonakdar S. (2016). Morphology, proliferation, and gene expression of gingival fibroblasts on laser-lok, titanium, and zirconia surfaces. Lasers Med. Sci..

[4] Renvert S., Polyzois I., Claffey N. (2011). How do implant surface characteristics influence peri-implant disease?. J. Clin. Periodontol..

[5] Rutkunas V., Bukelskiene V., Sabaliauskas V., Balciunas E., Malinauskas M., Baltriukiene D. (2015). Assessment of human gingival fibroblast interaction with dental implant abutment materials. J. Mater. Sci. Mater. Med..

[6] Schwarz F., Mihatovic I., Golubovic V., Eick S., Iglhaut T., Becker J. (2014). Experimental peri-implant mucositis at different implant surfaces. J. Clin. Periodontol..

[7] Lee D.W., Kim J.G., Kim M.K., Ansari S., Moshaverinia A., Choi S.H., Ryu J.J. (2015). Effect of laser-dimpled titanium surfaces on attachment of epithelial-like cells and fibroblasts. J. Adv. Prosthodont..

[8] Ramaglia L., Di Spigna G., Capece G., Sbordone C., Salzano S., Postiglione L. (2015). Differentiation, apoptosis, and GM-CSF receptor expression of human gingival fibroblasts on a titanium surface treated by a dual acid-etched procedure. Clin. Oral Investig..

[9] Schwarz F., Mihatovic I., Becker J., Bormann K.H., Keeve P.L., Friedmann A. (2013). Histological evaluation of different abutments in the posterior maxilla and mandible: An experimental study in humans. J. Clin. Periodontol..

[10] Kloss F.R., Najam-Ul-Haq M., Rainer M., Gassner R., Lepperdinger G., Huck C.W., Bonn G., Klauser F., Liu X., Memmel N. (2007). Nanocrystalline diamond—An excellent platform for life science applications. J. Nanosci. Nanotechnol..

[11] Burgers R., Gerlach T., Hahnel S., Schwarz F., Handel G., Gosau M. (2010). In vivo and in vitro biofilm formation on two different titanium implant surfaces. Clin. Oral Implants Res..

[12] Kloss F.R., Steinmüller-Nethl D., Stigler R.G., Ennemoser T., Rasse M., Hachl O. (2011). In vivo investigation on connective tissue healing to polished surfaces with different surface wettability. Clin. Oral Implants Res..

[13] Kloss F.R., Francis L.A., Sternschulte H., Klausner F., Gassner R., Rasse M., Bertel E., Lechleitner T., Steinmüller-Nethl D. (2008). Commercial developments of nan-crystalline diamond—Two prototypes as case studies. Diam. Relat. Mater..

[14] Kloss F.R., Gassner R., Preiner J., Ebner A., Larsson K., Hachl O., Tuli T., Rasse M., Moser D., Laimer K. (2008). The role of oxygen termination of nanocrystalline diamond on immobilisation of BMP-2 and subsequent bone formation. Biomaterials.

[15] Lechleitner T., Klauser F., Seppi T., Lechner J., Jennings P., Perco P., Mayer B., Steinmuller-Nethl D., Preiner J., Hinterdorfer P. (2008). The surface properties of nanocrystalline diamond and nanoparticulate diamond powder and their suitability as cell growth support surfaces. Biomaterials.

[16] Jakubowski W., Bartosz G., Niedzielski P., Szymanski W., Walkowiak B. (2004). Nanocrystalline diamond surface is resistant to bacterial colonization. Diam. Relat. Mater..

[17] Mekayarajjananonth T., Winkler S. (1999). Contact angle measurement on dental implant biomaterials. J. Oral Implantol..

[18] Kilkenny C., Browne W.J., Cuthill I.C., Emerson M., Altman D.G. (2010). Improving bioscience research reporting: The arrive guidelines for reporting animal research. J. Pharmacol. Pharmacother..

[19] Schwarz F., Herten M., Sager M., Wieland M., Dard M., Becker J. (2007). Histological and immunohistochemical analysis of initial and early subepithelial connective tissue attachment at chemically modified and conventional SLA^®^ titanium implants. A pilot study in dogs. Clin. Oral Investig..

[20] Mustafa K., Odén A., Wennerberg A., Hultenby K., Arvidson K. (2005). The influence of surface topography of ceramic abutments on the attachment and proliferation of human oral fibroblasts. Biomaterials.

[21] Kim H., Murakami H., Chehroudi B., Textor M., Brunette D.M. (2006). Effects of surface topography on the connective tissue attachment to subcutaneous implants. Int. J. Oral Maxillofac. Implants.

[22] Yamada K., Hoshina H., Arashiyama T., Arasawa M., Arai Y., Uoshima K., Tanaka M., Nomura S. (2011). Immediate implant loading following computer-guided surgery. J. Prosthodont. Res..

[23] Ebisawa K., Kato R., Okada M., Sugimura T., Latif M.A., Hori Y., Narita Y., Ueda M., Honda H., Kagami H. (2011). Gingival and dermal fibroblasts: Their similarities and differences revealed from gene expression. J. Biosci. Bioeng..

[24] Lee S., Goh B.T., Wolke J., Tideman H., Stoelinga P., Jansen J. (2010). Soft tissue adaptation to modified titanium surfaces. J. Biomed. Mater. Res. Part A.

[25] Berglundh T., Gotfredsen K., Zitzmann N.U., Lang N.P., Lindhe J. (2007). Spontaneous progression of ligature induced peri-implantitis at implants with different surface roughness: An experimental study in dogs. Clin. Oral Implants Res..

